# A quantitative study on the impact of a community falls pharmacist role, on medicines optimisation in older people at risk of falls

**DOI:** 10.1186/s12877-024-05189-6

**Published:** 2024-07-15

**Authors:** Paula Crawford, Rick Plumb, Paula Burns, Stephen Flanagan, Carole Parsons

**Affiliations:** 1 Medicines Optimisation Older People Pharmacy Team, Belfast Health and Social Care Trust, Belfast, UK; 2 School of Medicine Dentistry and Biomedical Sciences, Queen’s University Belfast, and Belfast Health and Social Care Trust, Belfast, UK; 3 Pharmacy Department, Musgrave Park Hospital, Belfast Health and Social Care Trust, Belfast, UK; 4 School of Pharmacy, Queen’s University Belfast, 97 Lisburn Road, Belfast, BT9 7BL UK

**Keywords:** Falls Risk increasing drugs (FRIDs), Medicines optimisation, Deprescribing, Polypharmacy, Older people

## Abstract

**Background:**

The World Falls guidance includes medication review as part of its recommended multifactorial risk assessment for those at high risk of falling. Use of Falls Risk Increasing Drugs (FRIDs) along with polypharmacy and anticholinergic burden (ACB) are known to increase the risk of falls in older people.

**Method:**

The impact of a community falls pharmacist within a hospital Trust, working as part of a multi-professional community falls prevention service, was evaluated in 92 people aged 65 years or older, by analysing data before and after pharmacist review, namely: number and type of FRIDs prescribed; anticholinergic burden score using ACBcalc^®^; appropriateness of medicines prescribed; bone health review using an approved too; significance of clinical intervention; cost avoidance, drug cost savings and environmental impact.

**Results:**

Following pharmacist review, there was a reduction in polypharmacy (mean number of medicines prescribed per patient reduced by 8%; *p* < 0.05) and anticholinergic burden score (average score per patient reduced by 33%; *p* < 0.05). Medicines appropriateness improved (Medicines Appropriateness Index score decreased by 56%; *p* < 0.05). There were 317 clinically significant interventions by the community falls pharmacist. One hundred and one FRIDs were deprescribed. Annual cost avoidance and drug cost savings were £40,689-£82,642 and avoidable carbon dioxide (CO_2_) emissions from reducing inappropriate prescribing amounted to 941 kg CO_2_.

**Conclusion:**

The community falls pharmacist role increases prescribing appropriateness in the older population at risk of falls, and is an effective and cost-efficient means to optimise medicines in this population, as well as having a positive impact on the environment.

## Background

In the United Kingdom (UK), people aged 65 years and older have the highest risk of falling, with around one-third of people aged 65 years and over and around half of people aged 80 years and over falling at least once a year [1]. Falls are listed as the number one reason for trauma in older people at emergency departments and are the leading cause of injuries that result in older people being admitted to hospital [2].

The World Falls guidance includes medication review as part of its recommended multifactorial risk assessment for those at high risk of falling [3]. Use of Falls Risk Increasing Drugs (FRIDs) [4], polypharmacy and anticholinergic burden (ACB) are known to increase the risk of falls in older people as outlined in the National Institute for Health and Care Excellence (NICE) guidance on falls risk assessment and prevention, which includes medication review as part of its recommended multifactorial risk assessment [5].

Problematic polypharmacy is defined as the prescribing of multiple medications inappropriately, or where the intended benefit of the medication is not realised [6]. Deprescribing is defined as the process of tapering, stopping, discontinuing or withdrawing drugs, with the goal of managing polypharmacy and improving outcomes [7, 8]. It is commonly reported that the risk of adverse drug events and likelihood of harm increase as the number of prescribed drugs increases [9]. In a longitudinal study conducted in England, Dhalwani et al. reported that using ten or more medicines was associated with a 50% higher rate of falls, using five or more medicines was associated with a 21% increased rate of falls, and using four or more medicines was associated with an 18% increased risk of falls compared to people without polypharmacy [10]. Other risk factors which are associated with falls include potential adverse effects of medication, and orthostatic hypotension. There is a well-developed evidence base that highlights the association between ACB and risk of fractures and falls, including reports that exposure to anticholinergic medication is associated with an increased risk of falls compared to patients not exposed to anticholinergics [11, 12].

For patients taking four or more medicines, risk of falling, fear of falling and cognitive impairment increase significantly [13]. In March 2016, the Northern Ireland (NI) Medicines Optimisation Quality Framework was published, outlining what patients could expect from their medicines in order to gain the best possible outcomes every time they were prescribed, dispensed or administered [14]. Since then, pharmacists have been developing and implementing new models of practice for older people throughout NI supported by this framework.

In 2021, a large Health and Social Care Trust in Northern Ireland appointed a pharmacist to work at the interface of primary and secondary care, as part of the multidisciplinary Community Falls Prevention service, to optimise medicines in older people at risk of falls. The community falls pharmacist receives referrals from healthcare professionals including primary care General Practitioners (GPs), and the Northern Ireland Ambulance Service (NIAS) for patients whose home they have been called out to, and those presenting to a Hospital Emergency Department with a fall, but not admitted to hospital. The pharmacist visits patients in their own homes to undertake a structured medication review in relation to FRIDs [4] and a bone health review [15]. In this study, we explored the impact of the role of a community falls pharmacist working as part of the multi-professional Community Falls Prevention Service on medicines optimisation in older people at risk of falls.

## Methods

This is a quantitative prospective study on the impact of a novel community falls pharmacist role, on medicines optimisation in relation to use of FRIDs [4] in older people who have had a fall.

Patients were reviewed at their home or by telephone (depending on factors such as patient preference and Covid restrictions) during a structured, in-depth medication review by the community falls pharmacist.

**Fig. 1:**
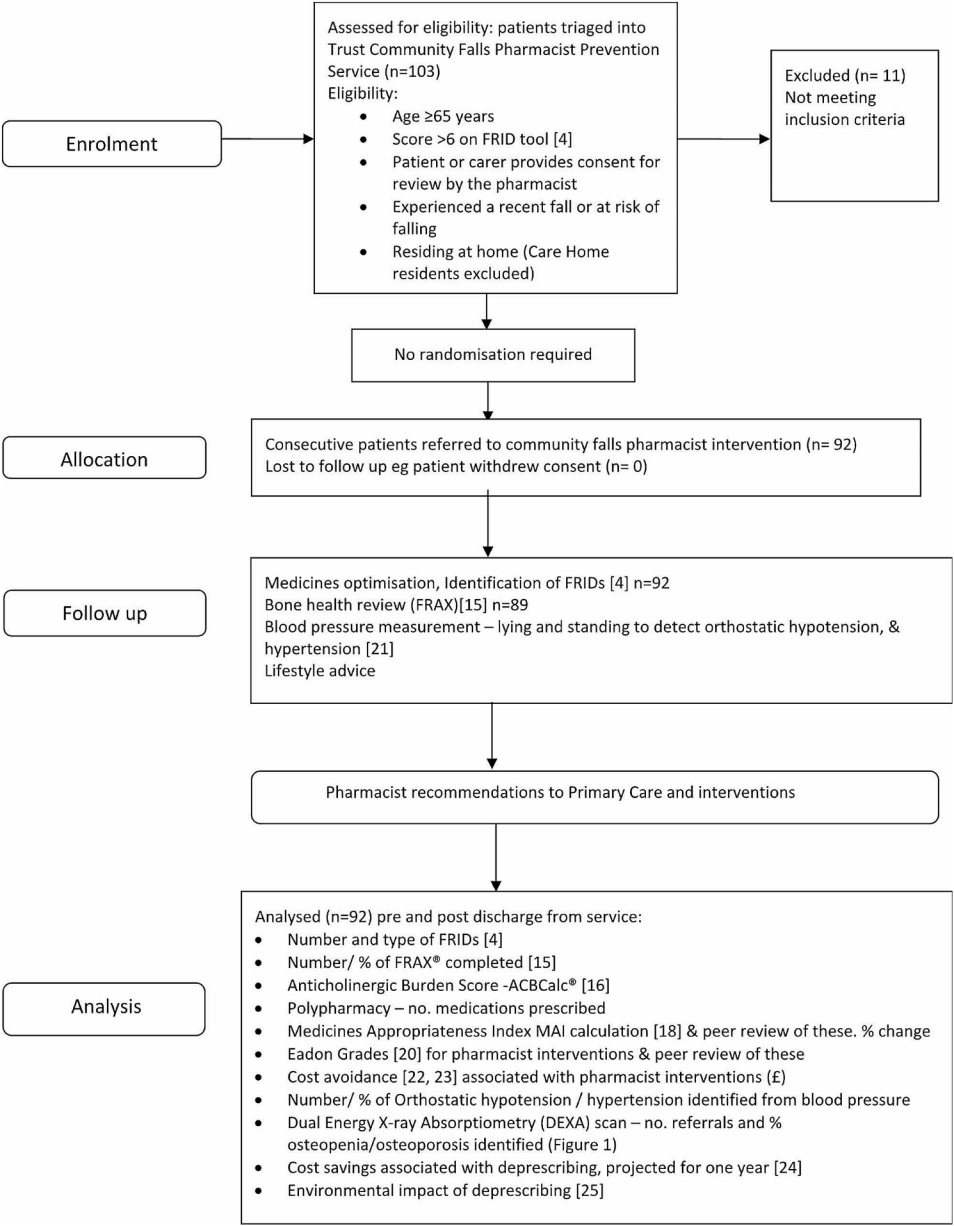
Evaluation of community falls pharmacist role

The process for inclusion in the study including inclusion criteria and review by the community falls pharmacist is outlined in Fig. 1.

Personal and Public Involvement (PPI) was sought in June 2022 from a consultative forum at Age NI, a charity providing a range of services, information and advice to older people in Northern Ireland.

Data were collected on admission and discharge from the community falls pharmacist service in relation to:

- Number and type of FRIDs [4] prescribed.
- Calculation of anticholinergic burden score using the ACB calculator (ACBcalc^®^) tool based on two different scoring systems, which have been demonstrated to show validity and reliability [16, 17].
- Polypharmacy and the appropriateness of medicines prescribed: The number of medications prescribed was recorded and the appropriateness of medication was measured using the Medicines Appropriateness Index MAI [18] before and after review by the community falls pharmacist. The MAI score [18] assigned by the community falls pharmacist was independently peer reviewed and verified by a lead clinical pharmacist. The clinimetrically derived MAI has demonstrated reliablity and validity and is widely used to measure potentially inappropriate prescribing (PIP) in older adults across a range of clinical settings [19]. Ten criteria were considered for each medication prescribed, with each criterion associated with a weighted score from 1 to 3, and a possible maximum total score of 18. The weighted MAI score per patient is calculated by adding the MAI scores for each drug in the patient’s regimen. Lower MAI scores indicate more appropriate prescribing.

- Clinical interventions: The quality of clinical interventions made by the pharmacist during the medicines optimisation review was measured using a six-point Eadon grading system [20] which identifies whether the intervention has led to an improvement in patient care, prevented major adverse reactions, or potentially saved a life, or conversely was detrimental to patient care. Grades ≥ 4 indicate a significant intervention resulting in improved standards of patient care. Clinical interventions included pharmacist recommendations to primary care GPs to reduce dosage of or de-prescribe medication carrying an associated falls risk. Many FRIDs, for example psychotropic agents, should not be stopped abruptly. These type of agents were gradually withdrawn or reduced to a stop where appropriate. Interventions also included recommendations to commence therapy to treat underlying conditions identified during the review, for example osteoporosis. Recommendations on deprescribing were agreed in collaboration with the patient and a summary letter sent to the GP.

**Fig. 2:**
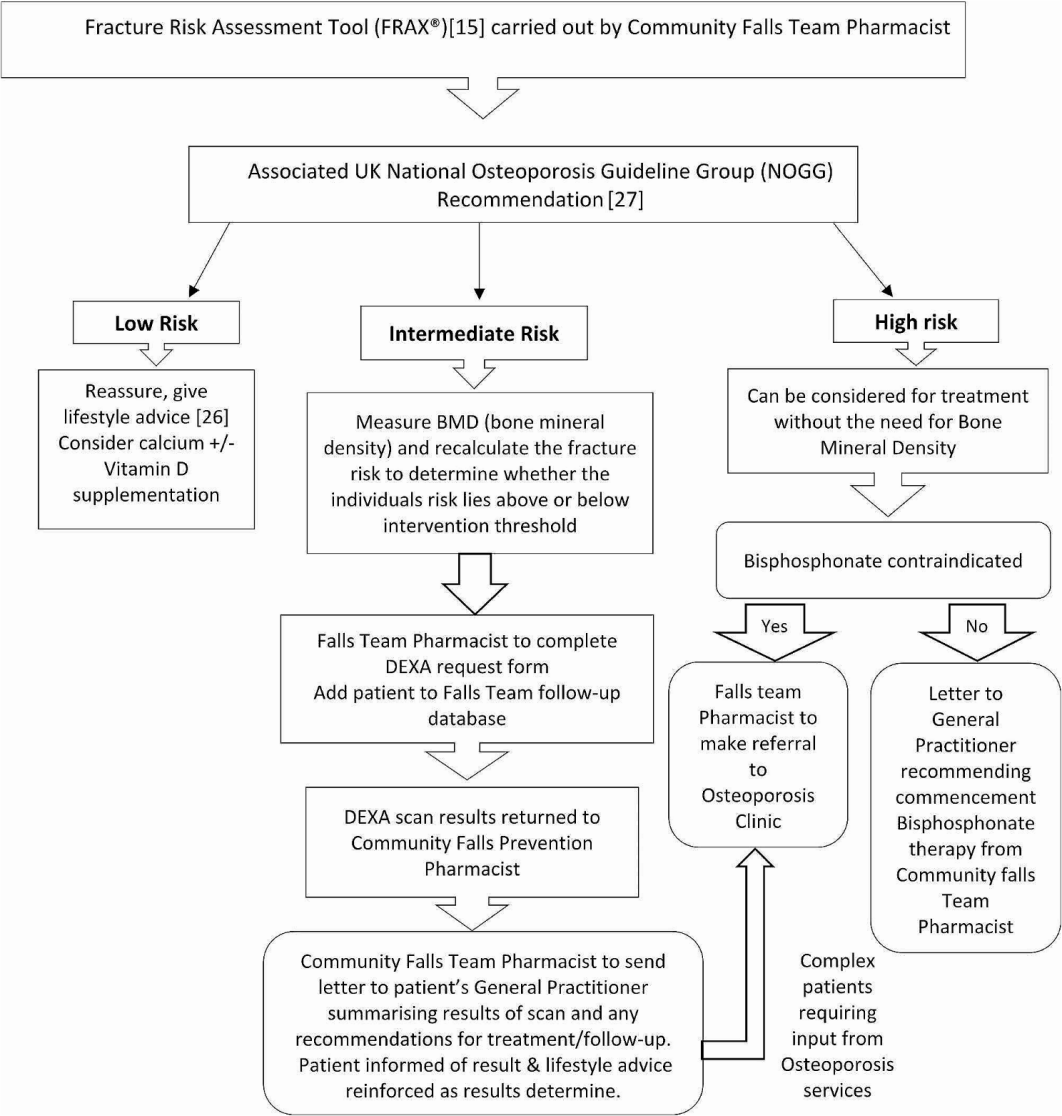
Hospital Trust Community Falls Prevention Service: Direct Pathway for internal referral from Community Falls Pharmacist to Trust Osteoporosis Services for Dual Energy X-ray Absorptiometry (DEXA) scan

The following were also undertaken by the pharmacist:

- Measurement of lying/ standing manual blood pressure to identify potential postural drop in blood pressure, hypotension and hypertension [21].
- Bone health review undertaken using an approved tool (FRAX^®^) [15] and outcomes:
  - Provision of lifestyle advice as required e.g. information leaflet supplied on bone health/dietary advice on calcium and vitamin D intake [26].
  - Commencement of medicines such as bisphosphonate, vitamin D or calcium if indicated [27].
- Referral of appropriate patients by the pharmacist for Dual-Energy X-ray Absorptiometry (DEXA) Bone Mineral Density (BMD) scan using a new direct referral pathway (Fig. 2), and quantitative assessment of the outcome of these as:

i. Normal: lifestyle advice.
ii. Osteopenia: commencement of vitamin D and/or calcium.
iii. Osteoporosis: commencement of bisphosphonate if indicated and onward referral to Trust Osteoporosis Service.

- Calculation of cost avoidance of pharmacist interventions was measured based on the School of Health And Related Research at Sheffield University (ScHARR) Tool [22], which defines the costs related to medication errors and Adverse Drug Events (ADEs). ScHARR cost avoidance estimates, inflated to 2022 prices using the Office for National Statistics (ONS), Consumer Price Index (CPI) health deflators, as updated by the iSimpathy Report 2023 [23], were applied.
- Cost savings from deprescribing potentially inappropriate medicines were calculated based on twelve months savings according to prices listed in the Northern Ireland Business Services Organisation, Drug Tariff September 2023 [24].
- Environmental impact over 12 months, associated with cost savings from medicines deprescribing was calculated based on the environmental impact report associated with the NICE guideline on medicines optimisation (NG5) 2015, which estimates that every pound (£) spent on pharmaceuticals generates greenhouse gas emissions of 0.1558 kg of carbon dioxide (CO_2_) [25].

### Statistical analysis

Demographic data were analysed and ranges, averages and medians calculated as appropriate. Names and numbers of FRIDs prescribed, clinical interventions and their grading, ACB [16], number of medicines prescribed, and MAI scores [18] before and after pharmacist review were entered into Microsoft Excel^®^ 2016 for analysis. Continuous data were tested for normality of distribution and inferential statistical tests were conducted as appropriate. Significance was set *a priori* at *p* ≤ 0.05.

Specific subgroups, for example those who had a FRAX^®^ [15] completed, or were referred for DEXA scan, were explored further with regard to outcomes.

## Results

Between June 2022 and August 2023, 103 patients were referred to the community falls pharmacist, and of these, 92 patients were reviewed by telephone (35%) or during home visit (65%). Patients were not reviewed if they did not meet the inclusion criteria or if they declined participation.

Patients’ ages ranged from 59 to 96 years; average age was 79 ± 7.9 years, and median age was 78 years. 25% of those reviewed were male and 75% female. All patients reviewed by the community falls pharmacist (*n* = 92) were taking one or more FRID(s) [4], and the average number of FRIDs per patient was four. The number of FRIDs prescribed per patient ranged from 1 to 8. A total of 367 FRIDs were identified and 101 were deprescribed. Table 1 outlines the top 10 FRIDs deprescribed.

**Table 1:** List of top 10 FRIDs deprescribed by community falls pharmacist

Medication Name	Number of patients (%)
Amitriptyline	21 (23)
Codeine	12 (13)
Furosemide	6 (7)
Tramadol	6 (7)
Mirtazapine	6 (7)
Solifenacin	5 (5)
Promethazine	4 (4)
Diazepam	3 (3)
Gabapentin	3 (3)
Pregabalin	3 (3)

### Anticholinergic burden (ACB)

Average ACB scores [16] significantly reduced from 4.2 before pharmacist intervention to 2.8 after medicines optimisation (↓ by 33%; *p* < 0.05).

### Polypharmacy and medicines appropriateness index (MAI)

Following pharmacist review, the mean number of medicines per patient reduced significantly from 10.4 to 9.6 (↓ by 8%; *p* < 0.05). The mean MAI [18] score pre-pharmacist review was 13.0; this reduced significantly to 5.7 post-review (↓ by 56%; *p* < 0.05), indicating that prescribing appropriateness had improved.

### Clinical interventions

Eadon grades, cost avoidance and environmental impact of pharmacist review are outlined in Table 2. There were 317 clinical interventions made by the community falls pharmacist, with an average of 3.4 interventions per patient.

**Table 2:** Eadon graded interventions by the community falls pharmacist (n *=* 317)

Eadon Grade [20]	Description	*n*	Examples from Pharmacist Medicine Reviews
3	Significant but does not lead to an improvement in patient care	1 (0.3%)	
4	Significant and results in an improvement in the standard of care	297 (93.7%)	Stopped prochlorperazine which is likely causing dry mouth and existing swallowing issues which will have significant adverse effect if persists in relation to nutrition, Quality of Life & unnecessary investigations for swallow Ongoing hyponatremia: amitriptyline may be a contributing factor to that along with fluoxetine high dose and thiazide diuretic
5	Very significant and prevents major organ failure or adverse reaction of similar importance	19 (6.0%)	Risedronate contraindicated in Creatinine Clearance (CrCl) < 30 ml/min
6	Potentially life-saving	0	

### Blood pressure

Twenty patients (22%) were identified as having orthostatic hypotension, uncontrolled hypertension or hypotension as a result of the community falls pharmacist review. The pharmacist provided advice to the patient along with a patient information leaflet on hydration and minimising postural drop where appropriate, and written recommendations were made to GPs, to review relevant medication such as anti-hypertensive medication and monitor blood pressure if required.

### Bone health review

**FRAX**^®^[15] assessment was undertaken in 89 patients (97%).

- Calcium and vitamin D initiation was recommended in 13 (15%) and 38 (43%) patients respectively, and oral bisphosphonate commenced in 10 (11%) patients [27].
- **DEXA outcome**: 31 patients (35%) were referred for DEXA scan; of these, four patients (13%) did not attend and declined a further appointment. Osteopenia and osteoporosis were newly identified in 17 patients (55%) and three patients (10%) respectively. Seven patients (23%) had a normal result.

**Other referrals** 20 patients (22%) were referred to other services including Psychiatry of Old Age, Diabetes Specialist Nurse and Urology.

Table 3 illustrates cost avoidance [22, 23], drug cost savings [24] for deprescribed FRIDs and environmental impact [25] of community falls pharmacist optimising medicines and use of FRIDs in older people over a 14-month period, and adjusted for 12 months.

Annual cost avoidance due to pharmacist interventions was in the range of £34,648 – £76,601 along with drug cost savings of £6041, amounting to total savings of £40,689-£82,642, and invest to save return of £1.25 to £2.54 for every £1 invested.

**Table 3:** Cost avoidance [22, 23], and environmental impact [25] of community falls pharmacist optimising medicines and use of FRIDs in older people over a 14-month period

Intervention description (Eadon criteria [20])	Cost avoidance ScHARR model [22, 23] £	Eadon Grade [20]	Number of interventions made by falls pharmacist (%)	Cost avoidance £
Potentially lethal	1334–2606	6	0	0
Potentially serious	877–1824	5	19 (6.0%)	16,663–34,713
Potentially significant	80–184	4	297 (93.7%)	23,760–54,648
Minor	0–7	1–3	1 (0.3%)	0–7
Total cost avoidance due to falls pharmacist interventions: 14 months = £40,423–89,368 adjusted for **12 months** = **£34,648–76,601**				
12-month projected savings (drug cost) for FRIDs deprescribed [24]				**£6041**
Total potential cost avoidance: community falls pharmacist 0.5WTE 8a salary 12 months = £32,564 ‘Invest to save’ return in range of £1.25 to £2.54 per £1 invested				£40,689-£82,642
Environmental impact of reducing inappropriate prescribing [25]: Avoidable CO_2_ emissions (kg) per 12 months				941 kg CO_2_

## Discussion

This study demonstrates the value of the community falls pharmacist role, based at the interface between primary and secondary care, in successfully deprescribing FRIDs within a community setting, in older people.

One hundred and one FRIDs were deprescribed as a result of the community falls pharmacist medicines optimisation. Amitriptyline was the most common FRID deprescribed, followed by codeine, furosemide, tramadol and mirtazapine. A range of other agents were deprescribed including antidepressants, sedatives, anti-epileptics and anti-psychotics. Onward referral by the pharmacist to other specialists including psychiatry of old age, osteoporosis services, urology services, and diabetes specialist nurse, highlights the complex nature of deprescribing in this group.

NICE recommends that all older people with recurrent falls or assessed as being at increased risk of falling should be considered for an individualised multifactorial intervention including a medication review with modification/withdrawal [5]. However, this is challenging due to the difficulty in identifying who is responsible and best placed to undertake medicines optimisation to reduce falls risk in older patients, as illustrated by studies highlighting the challenges in deprescribing in the hospital setting, including the artificial lifestyle of patients, and resource limitations [28–30]. Deprescribing in primary care settings is also not without challenges; studies exploring primary care physicians’ and patients’ views on deprescribing report barriers such as poor information sharing amongst organisations leading to hesitancy of primary care clinicians to deprescribe medicines prescribed by specialists in other care settings, competing demands on time, and patient-reported factors such as fear of withdrawal effects and lack of understanding around the decision to deprescribe [31–33].

In this study, the MAI [18] pre- and post-review reduced by an average of 56% (*p* < 0.05), indicating a significant improvement in appropriateness of prescribing. Deprescribing reduces the number of medicines prescribed, adverse drug reactions, and medicine costs, but to date, there has been little evidence of impact on clinical outcomes with the exception of falls [8, 34].

A number of tools have been developed, including implicit (judgment-based) and explicit (criterion-based) criteria to measure prescribing appropriateness, particularly for older patients with multimorbidity and polypharmacy [35–38]. In our study, we used the MAI tool [18], which uses implicit criteria and allows for the calculation of a total score, based on ten criteria including indication, effectiveness, dose, duration, correct directions, practical directions, drug-drug interactions, drug-disease interactions, duplication, and expense to evaluate the level of appropriateness or inappropriateness of prescribing. Although time consuming, the MAI was particularly useful in this research setting where an experienced pharmacist to apply clinical judgement and the full clinical record of the patient were available.

The explicit criteria that are widely used to address potentially inappropriate prescribing include the Beers Criteria [39], and the Screening Tool of Older Person’s Prescriptions/Screening Tool to Alert doctors to the Right Treatment (STOPP/START) Criteria [40], which were both updated in 2023. They are feasible to use in a busy environment and can be applied to any prescribed medication and large prescribing database, even if few clinical details are available. In relation to falls, the European Geriatric Medical Society published a Screening Tool of Older Persons Prescriptions in older adults with high fall risk (STOPPFall) [41], to support clinicians in the management of FRIDs and facilitate the deprescribing process, and is particularly useful to the community falls pharmacist in the setting we have described, and in healthcare settings where older people present with falls, in relation to optimising medicines in this patient group. The cause of falls is multifactorial in nature and a multidisciplinary approach to managing falls is recommended [3, 5, 13, 42], thus further development of specialist pharmacist roles such as the community falls role which we have shown to be effective in improving the appropriateness of medicines, along with the use of existing tools by prescribers to address inappropriate prescribing is a potential solution to support medicines optimisation in this older population. The cause of falls is multifactorial in nature and a multidisciplinary approach to managing falls is recommended [3, 5, 13, 42], thus further development of specialist pharmacist roles such as the community falls role which we have shown to be effective in improving the appropriateness of medicines, along with the use of the existing tools outlined above by prescribers to address inappropriate prescribing, is a potential solution to support medicines optimisation in this older population.

Anticholinergic burden scales were created as practical tools to identify the anticholinergic burden associated with medicines, which can in turn reflect the enhanced risk of falling with these medicines [9]. Medicines review by the community falls pharmacist had a statistically significant impact on reducing the ACB score, which is pertinent given that exposure to anticholinergic medication is associated with a higher risk of falls compared to those not exposed to anticholinergic medications [12].

The majority of clinical interventions by the pharmacist were determined to be Eadon grade 4 or above, indicating a significant intervention resulting in an improvement in care [20], including 6.0% preventing potentially serious outcomes; for example ceasing risedronate prescribing in an older woman with reduced creatinine clearance, where the risedronate is contra-indicated. Other examples include ceasing prochlorperazine therapy which was causing extreme dry mouth and exacerbating swallowing difficulties.

Twenty patients (22%) were identified as having orthostatic hypotension, uncontrolled hypertension or hypotension. Other interventions for this group included requesting a cardiology review, reducing ramipril, stopping amitriptyline and pramipexole, providing hydration and postural drop leaflet, referral to urology to consider stopping alpha blocker, and stopping furosemide.

Of those patients referred for DEXA scan, over half were newly identified as having osteopenia and three cases (10%) of osteoporosis were identified. An advantage of the DEXA referral pathway by the pharmacist (Fig. 2) is that it enables the pharmacist to refer patients directly without additional delays and workload to the GP in requesting the scan, or the Trust medical team in approving the request.

An invest to save return of £1.25 to £2.54 per £1 invested was realised by the pharmacist role. Although our focus was specifically on deprescribing of FRIDs alone, other studies such as an evaluation of consultant pharmacist case management of older people [43] also demonstrate savings associated with medicines optimisation as a whole in older people, yielding potential savings of £63-£144, 000 per annum associated with clinical interventions by the pharmacist and annual drug cost savings of £68,000.

Medicines account for 25% of CO_2_ emissions within the National Health Service in England each year [33, 44], and our study found that reduced inappropriate prescribing amounted to almost 1 tonne of avoidable CO_2_ emissions per year.

Another advantage of this pharmacist role at the interface between primary and secondary care is that it enables older people who have mobility issues and lack access to transport to receive a medicines optimisation review in their own homes. A study by Age UK found that the people with the worst health and lowest incomes struggle the most with travel to health services; 1.45 million of those aged 65 years and over in England found it difficult to travel to hospital, whilst 630,000 of those age 65 years and over found it difficult or very difficult to travel to their GP [45].

There is a lack of research specific to the Northern Ireland (NI) population, which has a different demographic and chronic disease burden compared to the rest of the UK as outlined in a 2016 burden of disease study, which identified notable differences such as the abundance of mental health conditions in NI and associated anxiety disorders [46]. Compared with a major international study by the Organisation for Economic Co-operation and Development (OECD), antidepressant usage in NI is reported as significantly higher than any of the other 23 countries surveyed, and antidepressant prescription rates in NI is cited as 129 daily doses per thousand, compared with the overall UK figure of 72 daily doses per thousand [47]. Many of the medicines used to treat anxiety disorders and mental health conditions also fall into the FRID category. Although withdrawal of FRIDs has been reported to be effective in reducing falls rates, the majority of older people do not have their medication reviewed or changed after a recent fall [42]. Systematic reviews and meta-analyses have identified psychotropic medicines including antidepressants such as tricyclic antidepressants, antipsychotics, benzodiazepines and cardiovascular medicines as the most important FRID classes [48–50].

There is a general paucity of published research in this area – specifically on the role of a pharmacist visiting patients in their own homes as part of a hospital-community interface post. Some small studies exist centered around the role of a community pharmacist based in a pharmacy retail premises, for example an implementation study establishing a fall prevention service was conducted in nine Dutch community pharmacies, where 91 patients received a medication review by the pharmacist, and medication adaptions were implemented for 32 patients [51]. Another small pilot study in Wisconsin, USA, used a targeted medication therapy management intervention on 38 older people, provided by one community pharmacist, which identified an important role for community pharmacists in modifying FRID use in this population [52].

There are a number of limitations to this present study. Firstly, the study was performed in a healthcare setting, within one hospital Trust, located in a densely populated area, which lends itself to healthcare professionals visiting clients within their own homes. This may reduce the application of the findings to other geographical areas, which are sparsely populated, for example in rural areas where from a practical perspective, travel time has to be taken into account in delivering the service. Secondly, hospital Trust non-medical prescribers such as the community falls pharmacist do not have routine access to make direct amendments to patients’ medication lists on their electronic care record in primary care due to Information Technology and governance restrictions. Therefore any prescription changes recommended by the community falls pharmacist are conveyed to the GP to enable amendments to be implemented. Ideally, in future a central electronic patient medication record and shared process will be developed to enable the community falls pharmacist to directly action recommendations in relation to prescribing. Thirdly, the study was not designed as a randomised controlled trial; therefore, the study design may be affected by selection and measurement bias. The use of a control group would have been ideal to evaluate this intervention in relation to the role of the pharmacist, and establish a cause-and-effect relationship between the service and the outcomes, however conducting a randomised controlled trial was not possible due to funding and time resource constraints. The present study provides useful evidence to inform further studies including randomised controlled trials.

This novel community falls pharmacist role offers a solution to the challenges of deprescribing FRIDs [4] in older people who have fallen. The outcomes of this research add to existing research and knowledge in this area and provide an insight into the role of a pharmacist where research is lacking, in relation to medicines and falls risk in older people living in their own homes within a community setting.

## Conclusion

An ageing population presents a growing challenge in relation to the demands and pressures on health and social care services. This study will potentially lead to improvements in how management of medicines in older people following a fall is optimised, and inform new pathways, policies and current practice. The community falls pharmacist role increases prescribing appropriateness in the older population at risk of falls, and it is an effective and cost-efficient means to optimise medicines in this population, as well as having a positive impact on the environment.

## Data Availability

The datasets used and/or analysed during the current study are available from the corresponding author on reasonable request. Data are not publicly available due to privacy and ethical restrictions.

## Abbreviations

ACB: Anticholinergic Burden
ADE: Adverse Drug Event
BMD: Bone Mineral Density
DEXA: Dual Energy X-ray Absorptiometry
ECR: Electronic Care Record
FRAX: Fracture Risk Assessment Tool
FRIDs: Falls Risk Increasing Drugs
GP: General Practitioner
MAI: Medicines Appropriateness Index
NI: Northern Ireland
NIAS: Northern Ireland Ambulance Service
NICE: National Institute of Health and Care Excellence (United Kingdom)
NOGG: National Osteoporosis Guideline Group (UK)
OECD: Organisation for Economic Co-operation and Development
ONS: Office for National Statistics
CPI: Consumer Price Index
PIP: Potentially Inappropriate Prescribing
PPI: Personal and Public Involvement
ScHARR: School of Health and Related Research (Sheffield University UK)
UK: United Kingdom
